# Apolipoprotein A1: a novel serum biomarker for predicting the prognosis of hepatocellular carcinoma after curative resection

**DOI:** 10.18632/oncotarget.12203

**Published:** 2016-09-23

**Authors:** Xiao-Lu Ma, Xing-Hui Gao, Zi-Jun Gong, Jiong Wu, Lu Tian, Chun-Yan Zhang, Yan Zhou, Yun-Fan Sun, Bo Hu, Shuang-jian Qiu, Jian Zhou, Jia Fan, Wei Guo, Xin-Rong Yang

**Affiliations:** ^1^ Department of Laboratory Medicine, Zhongshan Hospital, Fudan University, Shanghai, P. R. China; ^2^ Department of Liver Surgery, Liver Cancer Institute, Zhongshan hospital, Fudan University, Key Laboratory of Carcinogenesis and Cancer Invasion, Ministry of Education, Shanghai, P. R. China

**Keywords:** Apolipoprotein A1, hepatocellular carcinoma, serum biomarker, prognosis, circulating tumor cell

## Abstract

As a major protein constituent of high density lipoprotein, Apolipoprotein A1 (ApoA-1) might be associated with cancer progression. Our study investigated the serum ApoA-1 level for the prognosis of 443 patients with hepatocellular carcinoma (HCC) and its effects on tumor cells. We found that the serum ApoA-1 level was significantly lower in HCC patients with tumor recurrence, and was an independent indicator of tumor-free survival and overall survival. Low serum ApoA-1 levels were significantly associated with multiple tumors and high Barcelona Clinic Liver Cancer stage. The circulating tumor cell (CTC) levels were significantly higher in patients with low serum ApoA-1 compared with those with high serum ApoA-1 levels (4.03 ± 0.98 *vs.* 1.48 ± 0.22; *p*=0.001). In patients with detectable CTCs, those with low ApoA-1 levels had higher recurrence rates and shorter survival times. *In vitro* experiments showed that ApoA-1 can inhibit tumor cell proliferation through cell cycle arrest and promote apoptosis through down regulating mitogen-activated protein kinase (MAPK) pathway. In addition, ApoA-1 might impair extracellular matrix degradation properties of tumor cells. Taken together, our findings indicate that decreased serum ApoA-1 levels are a novel prognostic factor for HCC, and the role of ApoA-1 in inhibition of proliferation and promotion of apoptosis for tumor cells during their hematogenous dissemination are presumably responsible for the poor prognosis of patients with low ApoA-1 levels. Furthermore, AopA-1 might be a promising therapeutic target to reduce recurrence and metastasis for HCC patients after resection.

## INTRODUCTION

Hepatocellular carcinoma (HCC) is the most prevalent malignant disease with the second highest mortality rate worldwide [1]. To date, surgery remains the most effective treatment for HCC; however, the prognosis of HCC remains unsatisfactory because of high recurrence and metastasis post-surgery [2, 3]. The utility of conventional clinicopathological parameters, including histopathological characteristics, is insufficient for identifying patient subpopulations at high risk of recurrence and metastasis [4]. Therefore, there is an urgent requirement to develop novel biomarkers for identifying factors that predict tumor recurrence so that post-operative rational adjuvant treatments can be provided in a timely manner.

Apolipoprotein A1 (ApoA-1), which is encoded by a gene located on chromosome 11q23-q24, is a member of the apolipoprotein family [5, 6]. As a major protein constituent of high density lipoprotein, ApoA-1 plays an important role in protecting against cardiovascular diseases because of its anti-atherogenic function [7–11]. Recent studies indicated that ApoA-1 could inhibit the formation of tumor vessels [12], induce an anti-tumor immune microenvironment that prevents tumor progression [13] and serve as a potential therapeutic target for patients with cancer [14–16]. A pilot study found that the serum ApoA-1 level was significantly lower in patients with HCC [17, 18] and lower still in HCC patients with portal tumor thrombosis [19]. These findings implied that ApoA-1 might play a significant role in tumorigenesis and cancer progression of HCC. However, limited data were available on the clinical significance of serum ApoA-1 levels in HCC, and the mechanism of ApoA-1 involvement in HCC progression remained to be elucidated.

In this study, the serum ApoA-1 level was analyzed, and its clinical significance was evaluated in 433 HCC patients from two independent cohorts. Furthermore, the correlation between the ApoA-1 level and the circulating tumor cell (CTC) level was explored, and the effect of ApoA-1 on HCC cell lines was investigated. We found that the serum ApoA-1 level was significantly higher in patients with non-recurrent HCC and correlated with improved survival. Furthermore, a significant negative correlation between serum ApoA-1 and CTC levels was observed, and ApoA-1 could significantly inhibit the proliferation and promote the apoptosis of HCC cells *in vitro*. The serum ApoA-1 level could therefore serve as a useful prognostic marker for HCC, potentially reflecting the survival of tumor cells during their hematogenous dissemination.

## RESULTS

### Patient characteristics

The clinicopathologic characteristics of patients with HCC are summarized in Table 1. In the training cohort, 109 of 224 patients suffered recurrence after resection with a median follow-up time of 28.7 months (range 0.6-37.0 months) and 59 of 224 patients died before the last follow-up (median follow-up 33.0 months, range 3.0-37.0 months). The validation cohort comprised 77 of 219 patients with HCC with confirmed recurrence with a median follow-up time of 20.3 months (range 1.0-30.0 months), and 171 patients were still alive at a median follow-up of 23.0 months (range 3.6-30.0 months). All clinical characteristics were similar between the training and validation cohorts (Table 1).

**Table 1:** The clinicopathologic characteristics of patients in the training and validation cohorts

Characteristics		No. of patients	Training cohort		Validation cohort		*P*
			N=224	%	N=219	%	
Age (years)	≤50	172	85	37.95	87	39.73	0.770
	>50	271	139	62.05	132	60.27	
Sex	Women	73	33	14.73	40	18.26	0.370
	Men	370	191	85.27	179	81.74	
AFP, ng/mL	≤400	311	157	70.09	154	70.32	1.000
	>400	132	67	29.91	65	29.68	
ALT, U/L	≤75	412	211	94.20	201	91.78	0.355
	>75	31	13	5.80	18	8.22	
γ-GT, U/L	≤54	258	131	58.48	127	58.00	0.924
	>54	185	93	41.52	92	42.00	
HBsAg	Negative	58	28	12.50	30	13.70	0.779
	Positive	385	196	87.50	189	86.30	
Liver cirrhosis	No	108	51	22.77	57	26.03	0.440
	Yes	335	173	77.23	162	73.97	
No. of tumors	Single	362	180	80.36	182	83.11	0.464
	Multiple	81	44	19.64	37	16.89	
Tumor size, cm	≤5	272	141	62.95	131	59.82	0.558
	>5	171	83	37.05	88	40.18	
Tumor encapsulation	Complete	285	140	62.50	145	66.21	0.429
	None	158	84	37.50	74	33.79	
Satellite lesion	No	398	199	88.84	199	90.87	0.531
	Yes	45	25	11.16	20	9.13	
Vascular invasion	No	262	137	61.16	125	57.08	0.386
	Yes	181	87	38.84	94	42.92	
Tumor differentiation	I-II	283	142	63.39	141	64.38	0.844
	III-IV	160	82	36.61	78	35.62	
Child-Pugh score	A	409	210	93.75	199	90.87	0.287
	B	34	14	6.25	20	9.13	
BCLC stage	0+A	329	166	74.11	163	74.43	1.000
	B+C	114	58	25.89	56	25.57	

Abbreviations: AFP, α-fetoprotein; ALT, alanine aminotransferase; γ-GT, γ-Glutamyltransferase; HBsAg, hepatitis B surface antigen; BCLC, Barcelona Clinic Liver Cancer.

### Lower serum ApoA-1 level correlates with tumor recurrence and death of patients

**Figure 1:**
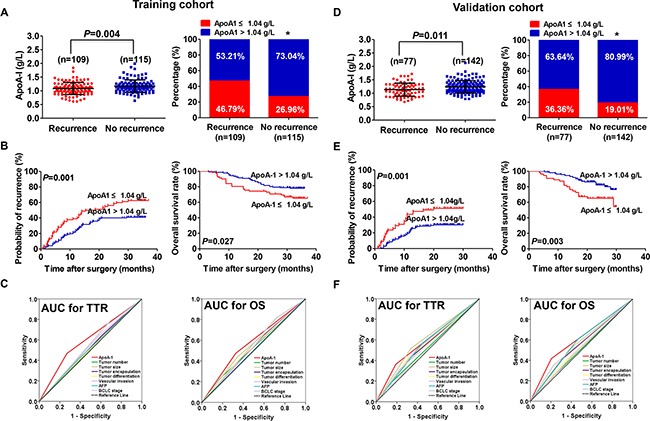
Prognostic significance of serum ApoA-1 levels in HCC patients underwent curative resection **A.** Distribution of serum ApoA-1 levels (left) and ApoA-1-positive rate (right) in recurrent and non-recurrent patients from the training cohort. **B.** Kaplan–Meier analysis for TTR (left) and OS (right) of patients with HCC according to serum ApoA-1 level in the training cohort. **C.** TTR (left) and OS (right) predictive ability of ApoA-1 was compared with other clinical parameters by ROC curves in the training cohort. **D.** Distribution of serum ApoA-1 levels (left) and ApoA-1-positive rate (right) in recurrent and non-recurrent patients from the validation cohort. **E.** Kaplan–Meier analysis for TTR (left) and OS (right) of patients with HCC according to serum ApoA-1 level in the validation cohort. **F.** TTR (left) and OS (right) predictive ability of ApoA-1 was compared with other clinical parameters by ROC curves in the validation cohort. “*” indicated *P* < 0.05.

We investigated the correlation between serum ApoA-1 level, tumor recurrence, and death of patients in the training cohort. The serum ApoA-1 level was significantly lower in HCC patients with recurrent disease compared with patients without recurrence (1.09 ± 0.02 g/L *vs*. 1.17 ± 0.02 g/L, *p*<0.05, Figure 1A). Using the optimal cutoff value (1.04 g/L, Supplementary Figure S1), a higher positive rate of patients with a low ApoA-1 level was observed in patients with tumor recurrence compared with patients without recurrence (46.79% vs. 26.96%, *p*<0.05, Figure 1A). Similarly, serum ApoA-1 level was significantly lower in patients who died (1.08 ± 0.03 g/L *vs.* 1.15 ± 0.02 g/L, *p*<0.05, Supplementary Figure S2A) and the positive rate of low ApoA-1 levels was significantly higher in patients who died in the validation cohort (47.46% *vs.* 32.73%, *p*<0.05, Supplementary Figure S2B).

### Prognostic value of serum ApoA-1 levels in the training cohort

Since the serum ApoA-1 level correlated with HCC recurrence and death, we further explored its prognostic significance in the training cohort. Using the X-tile 3.6.1 software, the optimal cutoff value (1.04 g/L) was set as to stratify patients into low ApoA-1 (≤1.04 g/L) and high (>1.04 g/L) groups (Supplementary Figure S1). Kaplan-Meier analysis showed that the time to recurrence (TTR) of HCC patients with a low serum ApoA-1 level was significantly shorter (median 17.1 months vs. not reached, *p*=0.001, Figure 1B) and had higher recurrence rates compared with those with a high ApoA-1 level (62.20% vs. 40.85%). Furthermore, the Overall survival (OS) of patients with HCC with a low serum ApoA-1 level was significantly shorter (*p*=0.027, Figure 1B) compared with those with a high ApoA-1 level (34.15% *vs.* 21.83%). In multivariate analysis, the serum ApoA-1 level was an independent indicator for TTR [Hazard ratio (HR), 0.39; 95% confidence interval (CI): 0.26-0.60; *p*<0.001; Table 2] and OS (HR, 0.47; 95% CI: 0.27-0.82; *p*=0.008; Table 2). The discrimination ability of ApoA-1 and clinical indices was compared by the area under curve (AUC) for TTR and OS (Figure 1C). The AUC for ApoA-1 was 0.60 (95% CI, 0.53–0.63; Sensitivity, 0.468; Specificity, 0.630; Youden's index, 0.098) for TTR, and 0.57 (95% CI, 0.49–0.66; Sensitivity, 0.475; Specificity, 0.673; Youden's index, 0.148) for OS, which was the strongest factor among indices (tumor number, tumor size, tumor encapsulation, tumor differentiation, vascular invasion, AFP, and BCLC stage) for predicting recurrence and survival in patients with HCC.

**Table 2:** Multivariate Cox regression analyses in the training and validation cohort

Variables	TTR		OS	
	HR(95% CI)	*P*	HR(95% CI)	*P*
**Training cohort**				
AFP, ng/ml (>400 vs ≤400)	1.048 (0.681-1.641)	0.832	0.548 (0.282-1.068)	0.077
γ-GT, U/L (>54 vs ≤54)	2.488 (1.654-3.743)	**<0.001**	1.930 (1.117-3.332)	**0.018**
No. of tumors, (multiple vs single)	0.856 (0.508-1.443)	0.559	1.065 (0.547-2.076)	0.852
Tumor size, cm (>5 vs ≤5)	0.861 (0.553-1.340)	0.507	1.483 (0.838-2.625)	0.176
Tumor encapsulation, (none vs complete)	1.250 (0.833-1.875)	0.281	1.196 (0.690-2.074)	0.524
Satellite lesion, (yse vs no)	0.907 (0.484-1.700)	0.761	0.776 (0.330-1.827)	0.562
Vascular invasion, (yes vs no)	0.735 (0.474-1.139)	0.169	0.707 (0.392-1.275)	0.249
Tumor differentation, (III-IV VS I-II)	1.014 (0.662-1.552)	0.950	1.102 (0.623-1.950)	0.738
ApoA-1, g/L (>1.04 vs ≤1.04)	0.394 (0.260-0.598)	**<0.001**	0.474 (0.273-0.824)	**0.008**
**Validation cohort**				
AFP, ng/ml (>400 vs ≤400)	1.205 (0.717-2.024)	0.481	1.199 (0.623-2.307)	0.588
γ-GT, U/L (>54 vs ≤54)	1.158 (0.731-1.834)	0.532	1.484 (0.821-2.685)	0.191
No. of tumors, (multiple vs single)	1.041 (0.576-1.881)	0.895	0.923 (0.427-1.994)	0.837
Tumor size, cm (>5 vs ≤5)	1.640 (0.974-2.760)	0.063	1.537 (0.796-2.969)	0.200
Tumor encapsulation, (none vs complete)	0.782 (0.485-1.261)	0.313	0.678 (0.371-1.238)	0.206
Satellite lesion, (yse vs no)	1.183 (0.581-2.411)	0.643	1.159 (0.464-2.894)	0.751
Vascular invasion, (yes vs no)	0.971 (0.577-1.636)	0.913	0.828 (0.427-1.604)	0.575
Tumor differentation, (III-IV VS I-II)	1.204 (0.743-1.953)	0.451	0.948 (0.508-1.769)	0.867
ApoA-1, g/L (>1.04 vs ≤1.04)	0.532 (0.326-0.868)	**0.011**	0.444 (0.243-0.772)	**0.010**

Abbreviations: AFP, α-fetoprotein; γ-GT, γ-Glutamyltransferase; ApoA-1, Apolipoprotein A1; TTR, time to recurrence; OS, Overall survival; HR, hazard ratio; CI, confidence interval.

**Figure 2:**
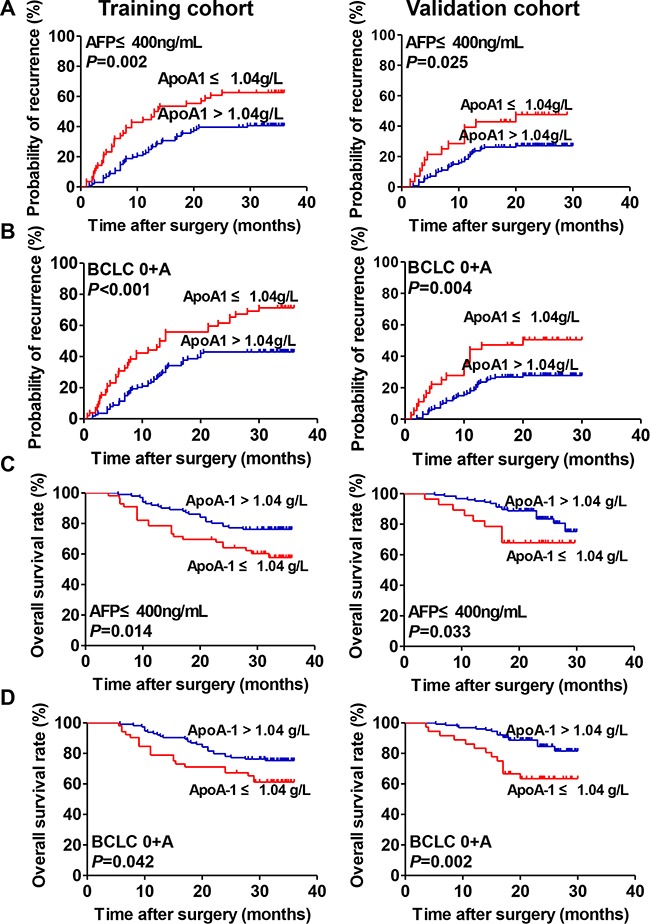
Prognostic significance of serum ApoA-1 levels of HCC patients in the low-risk and AFP ≤ 400 ng/mL subgroups **A.** Kaplan–Meier analysis of TTR of patients with HCC with AFP ≤ 400 ng/mL in the training (left) and validation (right) cohorts. **B.** Kaplan–Meier analysis of TTR of patients with HCC with BCLC stage 0+A in the training (left) and validation (right) cohorts. **C.** Kaplan–Meier analysis of OS of patients with HCC with AFP ≤ 400 ng/mL in training (left) and validation (right) cohorts. **D.** Kaplan–Meier analysis of OS of patients with HCC with BCLC stage 0+A in training (left) and validation (right) cohorts.

The prognostic significance of serum ApoA-1 level within conventional low-risk or α-fetoprotein (AFP) ≤ 400 ng/mL subgroups was further investigated. We found that serum ApoA-1 level was significantly associated with TTR and OS for the variables as follows: AFP ≤ 400 ng/mL for TTR (median 13.4 months *vs.* not reached, *p*=0.002, Figure 2A), Barcelona Clinic Liver Cancer (BCLC) stages 0+A for TTR (median 13.5 months *vs.* not reached, *p*<0.001, Figure 2B), AFP ≤ 400 ng/mL for OS (*p*=0.014, Figure 2C), BCLC 0+A for OS (*p*=0.042, Figure 2D).

### Prognostic value of serum ApoA-1 level in the validation cohort

The prognostic value of serum ApoA-1 level was further assessed using an independent cohort of 219 patients with HCC. The results were similar to those of the training cohort (Figure 1). A low serum ApoA-1 level was associated with shorter TTR (median 20.0 months *vs.* not reached, *p*=0.001, Figure 1E) and OS (*p*=0.003, Figure 1E). Cox regression analyses demonstrate that the serum ApoA-1 level was an independent indicator of TTR (HR, 0.53; 95% CI, 0.33-0.87; *p*=0.011; Table 2) and OS (HR, 0.44; 95% CI, 0.24-0.77; *p*=0.010; Table 2). Moreover, similar to the training cohort, the prognostic significance of the serum ApoA-1 level remained in the low-risk and AFP ≤400ng/mL subgroups (all *p*<0.05) (Figure 2). The discrimination ability of ApoA-1, as assessed by AUC, was 0.59 (95%CI, 0.53–0.62; Sensitivity, 0.364; Specificity, 0.810; Youden's index, 0.174) and 0.61 (95% CI, 0.54–0.66; Sensitivity, 0.417; Specificity, 0.795; Youden's index, 0.212) for TTR and OS (Figure 1F) respectively, which was higher than other clinical indexes.

### Correlation between the serum ApoA-1 level and clinical characteristics

Patients with HCC and a low serum ApoA-1 level were more likely to have multiple tumors (*p*=0.023, Table 3) and a high BCLC stage (*p*=0.007, Table 3) compared with those with a high serum ApoA-1 level. There was no significant difference in other clinical characteristics, including tumor size and vascular invasion (either micro or major) between the high and low serum ApoA-1-level groups in the training cohort. In the validation cohort, a low serum ApoA-1 level was associated with a high AFP level (*p*=0.001, Table 3) and a greater proportion of men (*p*=0.045, Table 3).

**Table 3:** Correlation between serum ApoA-1 levels and clinicopathologic characteristics

Clinical characteristics		Training cohort			Validation cohort		
		ApoA-1 ≤1.04g/L (N=82)	ApoA-1 >1.04g/L (N=142)	*P*	ApoA-1 ≤1.04g/L (N=55)	ApoA-1 >1.04g/L (N=164)	*P*
Age	≤50	32	53	0.886	20	67	0.634
	>50	50	89		35	97	
Sex	Female	9	24	0.248	5	35	**0.045^a^**
	Male	73	118		50	129	
AFP, ng/mL	≤400	56	101	0.653	28	126	**0.001**
	>400	26	41		27	38	
ALT, U/L	≤75	78	133	0.773^a^	50	151	0.780^a^
	>75	4	9		5	13	
γ-GT, U/L	≤54	55	76	0.050	35	92	0.348
	>54	27	66		20	72	
HBsAg	Negative	10	18	1.000	11	19	0.120
	Positive	72	124		44	145	
Liver cirrhosis	No	20	31	0.741	16	41	0.595
	Yes	62	111		39	123	
No. of tumor	Single	59	121	**0.023**	43	139	0.299
	Multiple	23	21		12	25	
Tumor size, cm	≤5	48	93	0.317	27	104	0.080
	>5	34	48		28	60	
Tumor encapsulation	Complete	47	93	0.253	34	111	0.510
	None	35	49		21	53	
Satellite lesion	No	70	129	0.271	49	150	0.594
	Yes	12	13		6	14	
Vascular invasion	No	46	91	0.257	27	98	0.208
	Yes	36	51		28	66	
Tumor differentation	I-II	47	95	0.195	30	111	0.103
	III-IV	35	47		25	53	
Child-Pugh score	A	76	134	0.775	50	149	1.000^a^
	B	6	8		5	15	
BCLC stage	0+A	52	114	**0.007**	36	127	0.107
	B+C	30	28		19	37	

Abbreviations: AFP, α-fetoprotein; ALT, alanine aminotransferase; γ-GT, γ-Glutamyltransferase; HBsAg, hepatitis B surface antigen; BCLC, Barcelona Clinic Liver Cancer; ApoA-1, Apolipoprotein A1.

a Fisher exact test.

### Correlation between the serum ApoA-1 level and CTC and its prognostic significance in HCC patients with detectable CTC

**Figure 3:**
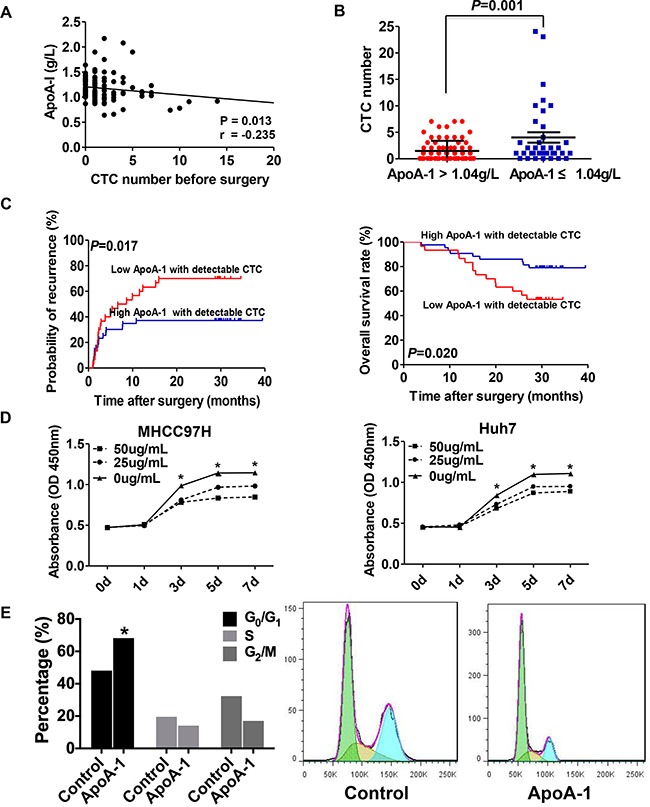
Correlation between serum ApoA-1 levels and CTC **A.** Correlation between serum ApoA-1 levels and the number of CTCs. **B.** CTC-positive rates of HCC patients with different serum ApoA-1 levels. **C.** The Kaplan–Meier analysis of TTR (left) and OS (right) for serum ApoA-1 levels in patients with detectable CTC. **D.** Cell viability was significantly inhibited by 25 or 50 ug/mL ApoA-1 compared with control group in MHCC97H (left) and Huh7 (right) cell lines. **E.** ApoA-1 treatment could effectively result in G0/G1 arrest in MHCC97H. “*” indicated *P* < 0.05.

The correlation between serum ApoA-1 and CTC levels was further investigated. Scatter-plot analyses revealed a significant negative correlation between serum ApoA-1 and CTC levels (r=–0.235; *p*=0.013; Figure 3A). The levels of CTC were significantly higher in patients with a low ApoA-1 level compared with those with a high serum ApoA-1 level (4.03 ± 0.98 *vs.* 1.48 ± 0.22; *p*=0.001; Figure 3B).

In light of the close relationship between CTC and serum ApoA-1 levels, we further explored the prognostic significance of serum ApoA-1 level in subgroups of patients presenting with CTC. In patients with detectable CTC, ApoA-1 levels ≤ 1.04 g/L were associated with higher recurrence rates (70.0% *vs.* 37.2%; *p*<0.05) and a shorter TTR (median, 7.6 months *vs.* not reached; *p*=0.017) compared with patients with ApoA-1 > 1.04 g/L (Figure 3C). In terms of OS, we also found that overall survival rate was significantly lower in patients with low ApoA-1 levels than those with high ApoA-1 levels (*p*=0.020, Figure 3C).

### ApoA-1 inhibits tumor cell proliferation, induces apoptosis and impairs their extracellular matrix degradation properties

To explore the effect of ApoA-1 on tumor cells, cell proliferation was analyzed. Compared with negative control (i.e., tumor cells treated without ApoA-1), tumor cell viability with ApoA-1 was significantly suppressed in a time-dependent manner after exposure to 25 or 50 μg/mL ApoA-1 for 7 days (0.834 ± 0.004 *vs.* 1.142 ± 0.042 for 5 days in MHCC97H, *p*<0.05;0.870 ± 0.008 *vs.* 1.098 ± 0.012 for 5 days in Huh7, *p*<0.05, Figure 3D). Further cell cycle tests were performed on MHCC97H cells and results showed that the percentage of cells in G_0/_G_1_ phase increased while the percentage of cells in S and G_2_/M phase decreased after ApoA-1 treatment (Figure 3E), which indicated cell cycle blocking function of ApoA-1.

**Figure 4:**
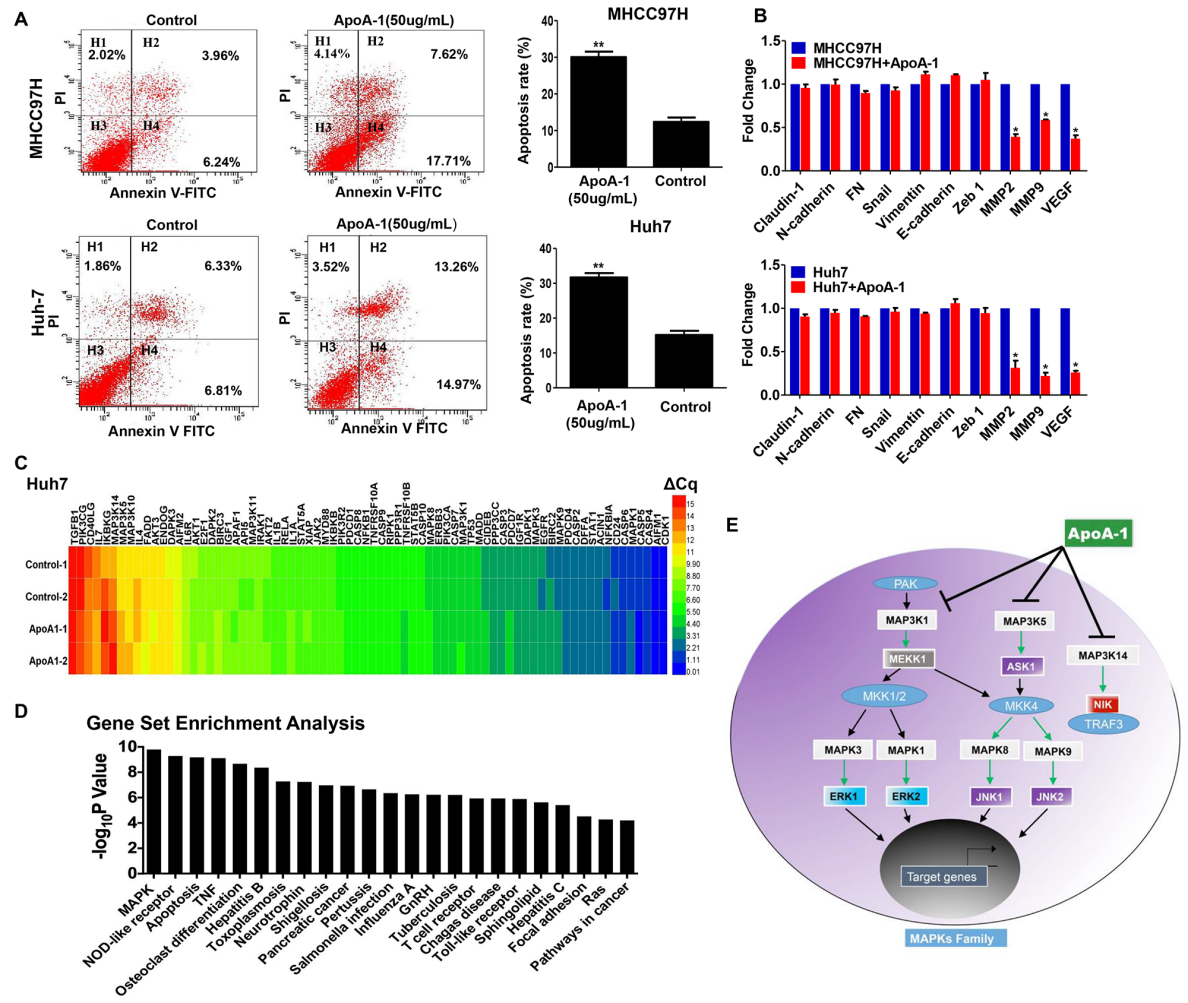
ApoA-1-induced apoptosis was associated with mitogen-activated protein kinase (MAPK) pathway **A.** Apoptosis was detected by annexinV-FITC/propidiumiodide staining. In MHCC97H (up) and Huh7 (below) cells, the ApoA-1-induced apoptosis rates were higher than control group (*P < 0.05, **P < 0.01). **B.** Relative expression of invasion-related genes after ApoA-1 treatment in MHCC97H (up) and Huh7 (below) cell lines. **C.** Heatmap of the results of PCR-array. **D.** Gene set enrichment analysis of altered genes involved in PCR array and results showed that MAPK signaling pathway was the most significant pathway affected by ApoA-1 treatment. **E.** Potential mechanism underlying ApoA-1 inducing apoptosis. ApoA-1 treatment could decrease the expression of up-stream molecules as well as down-stream molecules in MAPK pathway, and thus greatly induces the apoptosis of HCC cells.

The effect of ApoA-1 on tumor cell apoptosis was also investigated. We found that ApoA-1 treatment-induced apoptosis rates were 30.06 ± 1.51% and 21.67 ± 1.25% in MHCC97H and Huh7 cells, while the apoptosis rates were 12.34 ± 1.21% and 15.17 ± 1.18% in those without ApoA-1 treatment (*p*<0.001, Figure 4A).

Furthermore, we performed RT-PCR to observe expression changes of several invasion-related genes. Results showed that expression of epithelial-mesenchymal transition-related (EMT-related) genes showed no difference before and after ApoA-1 treatment. However, matrix metalloproteinases (MMPs) including MMP-2 and MMP-9 showed significant decrease after ApoA-1 treatment in both two cell lines, which indicating ApoA-1 might inhibit CTC formation via impairing extracellular matrix degradation properties of tumor cells. In addition, we also observed a dramatic decrease of vascular endothelial growth factor (VEGF) mRNA, which was in accordance with a previous study [12] (Figure 4B).

### ApoA-1-induced apoptosis was associated with mitogen-activated protein kinase (MAPK) pathway

Next, to further address how ApoA-1 induced apoptosis in HCC cells, a human apoptosis PCR-array was used to determine the expression of apoptosis –regulatory genes in Huh7 cells. A total of 88 genes were tested for screening potential pathway, For the sake of reliability, 74 genes with a Cq value less than 35 were further selected for comparison. When 1.50 was set as the cutoff for changes of expression (ApoA-1 treated versus untreated), the expressions of 14 genes were significantly altered by ApoA-1 treatment. Among these 14 genes, the expression of three pro-apoptotic genes including caspase 5 (CASP5), tumor necrosis factor receptor superfamily member 10B (TNFRSF10B), and apoptotic protease activating factor-1 (AFAP-1) were up-regulated (fold change: 1.62, 1.53, and 1.52, respectively). Meanwhile, the expressions of 11 anti-apoptotic genes including MAPK1 (also known as ERK2), MAPK3 (also known as ERK1), and Inhibitor of nuclear factor Kappa-B kinase subunit Gamma (IKKG) were found to be notably reduced after ApoA-1 treatment (Figure 4C). Details were listed in Supplementary Table S2.

We further conducted the Gene Set Enrichment Analysis (GSEA) to figure out the signaling pathways significantly altered by ApoA-1treatment to investigate the mechanism underlying ApoA-1-induced-apoptosis. MAPK signaling pathway was identified to be the most significant pathway with the smallest P value, and 9 of 14 altered genes were involved in this pathway (Figure 4D, Table 4). We further performed RT-PCR assay with different primers to determine the expression status of these 9 genes in Huh7 and MHCC97H, and the results were accordance with PCR-array data (Supplementary Figure S3).

**Table 4:** Gene set enrichment analysis of PCR-array

Pathway	Gene number	Genes	P value	Q value
MAPK	9	IKBKG, MAP3K14, MAPK9, MAP3K5, MAPK8, MAPK1, IL1A, MAP3K1, MAPK3	1.64E-10	2.34E-10
NOD-like receptor	7	IKBKG, MAPK9, MAPK8, MAPK1, MAPK3, BIRC3, CASP5	5.24E-10	2.49E-10
Apoptosis	10	IKBKG, MAP3K14, MAPK9, MAP3K5, MAPK8, MAPK1, MAPK3, BIRC3, APAF1, TNFRSF10B	6.76E-10	3.85E-13
TNF	8	IKBKG, MAP3K14, MAPK9, MAP3K5, MAPK8, MAPK1, MAPK3, BIRC3	7.83E-10	1.49E-10
Osteoclast differention	7	IKBKG, MAP3K14, MAPK9, MAPK8, MAPK1, IL1A, MAPK3	2.18E-09	2.49E-08
Hepatitis B	7	IKBKG, MAPK9, MAPK8, MAPK1, MAP3K1, MAPK3, APAF1	4.44E-09	4.22E-08
Toxoplasmosis	6	IKBKG, MAPK9, MAPK8, MAPK1, MAPK3, BIRC3	5.35E-08	4.22E-07
Neurotrophin	6	MAPK9, MAP3K5, MAPK8, MAPK1, MAP3K1, MAPK3	5.92E-08	4.22E-07
Shigellosis	5	IKBKG, MAPK9, MAPK8, MAPK1, MAPK3	1.09E-07	6.74E-07
Pancreatic cancer	5	IKBKG, MAPK9, MAPK8, MAPK1, MAPK3	1.18E-07	6.74E-07
Pertussis	5	MAPK9, MAPK8, MAPK1, IL1A, MAPK3	2.26E-07	1.17E-06
Salmonella infection	5	MAPK9, MAPK8, MAPK1, IL1A, MAPK3	4.51E-07	1.98E-06
Influenza A	6	MAPK9, MAPK8, MAPK1, IL1A, MAPK3, TNFRSF10B	5.62E-07	2.21E-06
GnRH	5	MAPK9, MAPK8, MAPK1, MAP3K1, MAPK3	5.99E-07	2.21E-06
Tuberculosis	6	MAPK9, MAPK8, MAPK1, IL1A, MAPK3, APAF1	6.21E-07	2.21E-06
T cell receptor	5	IKBKG, MAP3K14, MAPK9, MAPK1, MAPK3	1.17E-06	3.51E-06
Chagas disease	5	IKBKG, MAPK9, MAPK8, MAPK1, MAPK3	1.17E-06	3.51E-06
Toll-like receptor	5	IKBKG, MAPK9, MAPK8, MAPK1, MAPK3	1.28E-06	3.66E-06
Sphingolipid	5	MAPK9, MAP3K5, MAPK8, MAPK1, MAPK3	2.38E-06	6.17E-06
Hepatitis C	5	IKBKG, MAPK9, MAPK8, MAPK1, MAPK3	3.96E-06	9.81E-06
Focal adhesion	5	MAPK9, MAPK8, MAPK1, MAPK3, BIRC3	3.05E-05	5.79E-05
Ras	5	IKBKG, MAPK9, MAPK8, MAPK1, MAPK3	5.34E-05	8.70E-05
Pathways in cancer	6	IKBKG, MAPK9, MAPK8, MAPK1, MAPK3, BIRC3	6.43E-05	0.0001

Abbreviations: MAPK, mitogen-activated protein kinase; TNF, tumor necrosis factor; IKBKG, I-Kappa-B Kinase Subunit Gamma; BIRC3, Baculoviral IAP Repeat Containing 3; CASP5, caspase 5; IL1A, interleukin 1 alpha

## DISCUSSION

ApoA-1, which is generally considered to protect against cardiovascular disease, has been reported to exhibit a unique effect on tumor progression in recent studies [12, 13, 21, 22]. ApoA-1 was reported to be an early diagnosis marker in HCV-background HCC and low level of ApoA-1 was correlated with formation of portal vein tumor thrombus as well as the progression of HCC. However, by far, no specific data about the correlation between ApoA-1 level and TTR/OS was available and the prognostic value of serum ApoA-1 in HCC remained elusive [18, 19]. Our present study explored the clinical significance of serum ApoA-1 levels in HCC patients, and the potential mechanism mediated by ApoA-1 in tumor progression. We found that the serum ApoA-1 level was significantly lower in HCC patients with recurrent disease compared with patients without recurrence, and serum ApoA-1 was an independent predictor for recurrence and survival in HCC patients post-surgery in two independent cohorts. Considering the convenience of serum ApoA-1 analysis, there is potential for ApoA-1 to be used as a marker for tumor recurrence and treatment response surveillance. This may provide a powerful test, enabling accurate and early decision-making to tailor the most effective therapy according to the characteristics of individual tumors.

Hematogenous spread is an important cause of recurrence and metastasis in HCC, and CTC in the bloodstream plays an important role in HCC metastasis [23, 24]. A negative correlation was observed between the serum ApoA-1 level and the peripheral CTC level in HCC patients, and the recurrent rate of patients with high serum ApoA-1 levels was decreased significantly in HCC patients with detectable CTC. EMT is considered as a major mechanism involved in the dissemination of tumor cells in HCC [25], However, our current data showed that ApoA-1 treatment might inhibit CTC formation via EMT-independent mechanism, in which the expressions of MMP2, MMP9, and VEGF were strongly down-regulated (Figure 4B). These data suggested ApoA-1 might inhibit CTC formation via impairing extracellular matrix degradation properties of tumor cells and decreasing angiogenesis, which highlighted the potential mechanism of ApoA-1 in inhibiting tumor cells dissemination. Moreover, our vitro experiments implied that ApoA-1 could inhibit tumor cell proliferation and induce apoptosis. Of note, apoptosis-inducing function of ApoA-1 could be attributed to the inhibition of MAPK pathway, which plays a critical role in the carcinogenesis, maintenance and progression of HCC (Figure 4E) [20]. These results demonstrated the preliminary mechanism underlying the inhibition effect of ApoA-1 on HCC, which might provide a novel insight into anti-HCC research. Further systematical investigation focusing on the detailed mechanism of ApoA-1 in inactivating MAPK signaling is also undergoing in our lab.

Releasing of tumor cells into circulation is a multiple-step procedure, which including a great number of proteins, and ApoA-1 acts as one of the major molecules involved in this procedure, which could effectively affect the viability of CTCs. Based on our data, we believed that ApoA-1 was a significant negative regulator of CTC (*p*=0.013), though the correlation was a little bit weak (r=−0.235). Taken together, these findings indicated that ApoA-1 protein might decrease the CTC level by preventing CTC formation, affecting the survival, and inducing apoptosis of tumor cells in circulation, which resulted in higher recurrence rates and poor outcomes in HCC patients with low serum ApoA-1 levels. Therefore, improving the serum ApoA-1 level in HCC patients might be a promising therapeutic strategy to limit tumor recurrence and metastasis in HCC patients post-surgery.

In clinical practice, it is challenging to predict tumor relapse in low recurrence risk HCC subgroups. The present study is the first to show that preoperative serum ApoA-1 levels retain their prognostic value in those subgroups at risk for which conventional clinicopathological variables offer limited information predicting tumor recurrence. To date, AFP has been the most extensively used biomarker for diagnosis and surveillance in HCC patients [26–28]. However, it is difficult to monitor recurrence in the 30%-40% of HCC patients with low AFP levels [4, 28]. Here, we have also shown that determination of the preoperative serum ApoA-1 level is a promising and feasible tool for recurrence prediction in patients with a low AFP concentration. The predictive significance of the serum ApoA-1 level in those subgroups would be that clinicians could identify patients at high risk of recurrence and enable targeted rational adjuvant therapy post-surgery. Up to now, analysis of serum ApoA-1 levels has been performed as a routine clinical liver function test using commercially available kits. The detection of serum ApoA-1 levels can be easily standardized to provide accuracy and reproducibility, strengthening the practical value of this test in contributing information for early decision making regarding the most effective therapy for each HCC patient.

In this study, we enrolled an independent cohort of patients to validate the clinical utility of serum ApoA-1 levels and found that the clinical characteristics of the training and validation cohorts were similar, which indicated the reliability and universality of our findings. However, there are still several limitations to the present study. In our study, some recognized factors, such as tumor size, AFP, and vascular invasion are not the independent risk factors for TTR and OS for HCC. It might due to relatively short follow-up time and small size of cohort. Furthermore, it also should be noted that most HCC patients in China have a hepatitis B virus-positive background. Hence, a large-cohort, multicenter, long-term study including patients from different background is needed to validate the prognostic significance of the serum ApoA-1 level, and this is currently undergoing in our center. In addition, a comprehensive investigation of the mechanism of action of ApoA-1 on HCC cells also needs to be performed.

To our knowledge, this is the first report to demonstrate the clinical significance of serum ApoA-1 levels for predicting the prognosis of HCC patients. Our data indicates that the serum ApoA-1 level can serve as a novel, independent predictor of TTR and OS for HCC patients undergoing resection. Moreover, continuous monitoring of the serum ApoA-1 levels in HCC patients might provide useful information for the management of HCC and facilitate the implementation of different treatment options. The role of ApoA-1 in inhibiting the proliferation of tumor cells, as well as promoting apoptosis of cancer cells during tumor cell hematogenous dissemination, are presumably responsible for the high recurrence rate and poor prognosis observed in HCC patients with a low serum ApoA-1 level. The low cost, easy determination and reproducibility of serum ApoA-1 analysis make ApoA-1 a promising biomarker for assessing HCC prognosis in future clinical practice. Furthermore, improving the serum AopA-1 level in HCC patients might be a promising therapeutic strategy to reduce recurrence and metastasis for HCC patients undergoing resection.

## MATERIALS AND METHODS

### Study design

From January to July 2012, we recruited 224 patients with HCC who underwent curative resection at Zhongshan Hospital (Fudan University, Shanghai, China) as a training cohort. We next recruited a validation cohort of 219 patients with HCC who underwent resection from August 2012 to August 2013 (Table 1). From July 2010 to June 2011, 113 HCC patients undergoing curative resection were prospectively recruited and their previously reported CTC levels were analyzed [29]. HCC was defined according to the findings of imaging scans and biochemical assays, and diagnosis was confirmed by histopathology according to the criteria of the American Association for the Study of Liver Diseases guidelines. The stage of HCC was determined according to the BCLC guidelines [3]. Tumor differentiation was defined using the Edmondson grading system [30]. A blood sample (7.5 mL) used for CellSearch analysis was collected two days before resection as described in our previous report [29]. Ethical approval for the use of human subjects was obtained from the Research Ethics Committee of Zhongshan Hospital, and informed consent was obtained from each patient enrolled in the study.

### Follow-up and tumor recurrence

Every 3 to 4 months following resection, patients were monitored for serum AFP levels, and abdomen ultrasonography and chest X-rays were performed as previously described [31]. Follow-up ended in February 2015. TTR was defined as the interval between surgery and any diagnosed intrahepatic or extrahepatic recurrence. OS was defined as the interval between the date of surgery and the date of death, or the interval between surgery and the last observation.

### Determination of the serum ApoA-1 level

The serum ApoA-1 concentrations were determined by immunoturbidimetry, using Hitachi 7600 automated biochemistry analyzer and ApoA-1 assay kits (Diasys Diagnostic Inc., Holzheim, Germany) according to the manufacturer's protocol. Serum samples were collected two days before surgery, and stock at −80°C until detection. The X-tile 3.6.1 software (Yale University, New Haven, CT, USA) was used to determine the cutoff value of the serum ApoA-1 level for predicting tumor recurrence in the training cohort [32]. The optimal cut-off value was determined based on ApoA-1 concentrations, outcomes and time interval. Results from X-Tile analysis revealed an optimal cutoff point for the serum ApoA-1 level at 1.04 g/L in the training cohort (Supplementary Figure S1). Thus, patients were stratified into ApoA-1 high (>1.04 g/L) or low (≤1.04 g/L) groups for all subsequent analyses.

### Detection of CTC

The CTC detection was performed using the CellSearch system as previously described [33]. In brief, the semiautomated CellSearch platform (Janssen Diagnostics) enriches the sample for cells expressing EpCAM with ferromagnetic beads. Afterwards, fluorescently labeled monoclonal antibodies specific for leukocyte (CD45) and cytokeratins are used to distinguish epithelial cells from leukocytes. The identification and enumeration of CTCs were performed with the use of the CellSpotter Analyzer, and the results were expressed as the number of cells per 7.5 mL of blood.

### Cell lines and reagents

Two human HCC cell lines with different metastasis potential were used in this study. MHCC97H was obtained from our institute [34]. The Huh7 cell line was provided by the Cell Bank at the Institute of Biochemistry and Cell Biology, China Academy of Science (Shanghai, China). All cell lines were maintained in high-glucose Dulbecco's modified Eagle's medium (DMEM) supplemented with 10% heat-inactivated fetal bovine serum (FBS), 100 units/mL penicillin, and 100 mg/mL streptomycin at 37°C in a humidified incubator under 5% carbon dioxide. The recombinant human ApoA-1 (rhApoA1, *E. coli* source, #350-11, Peprotech, Connecticut, USA) was dissolved in phosphate-buffered saline to prepare a stock solution and was stored at −4°C.

### Cell proliferation and apoptosis assays

Cell proliferation and apoptosis assays were performed as previously described [35] with slight modifications. All experiments were performed in triplicate.

For the proliferation assay, cells were aliquoted into a 96-well plate at 1000/100 μL per well, incubated for 24 h, and treated with different concentrations of recombinant ApoA-1 (0, 25 or 50 ug/mL) in 200 μL DMEM containing 10% FBS. After 72 h, the medium was replaced by complete medium containing different concentrations of ApoA-1. Cells were exposed to ApoA-1 for 7 days. At the indicated time points, 20 μL of cell counting kit 8 (CCK-8) solution (Dojindo, Kamimashiki-gun, Kumamoto, Japan) was added to determine the number of viable cells in each well.

Cell apoptosis was analyzed by flow cytometry using annexin V-fluorescein isothiocyanate (FITC). Apoptosis Detection Kits (BD Biosciences, San Jose, CA, USA) were employed according to the manufacturer's protocol [35]. Briefly, cells were treated with 50 μg/mL ApoA-1 for 72 h, then harvested and suspended in a binding buffer (1×). Cells were similarly treated without ApoA-1 as a negative control. An aliquot of 100 μL was incubated with 5 μLannexin V-FITC and 5 μLpropidium iodide for 15 min in the dark, and 400 μL binding buffer (1×) was added to each sample. The stained cells were analyzed by flow cytometry within 1 h.

### RNA isolation, qRT-PCR and PCR array

Total RNA was extracted from cell lines using RNeasy mini kit (Qiagen, Germany) according to the manufacturer's instructions. mRNA expression in HCC cell lines was measured by qRT-PCR using an LightCycle480 instrument (Roche Diagnostics, Germany). qRT-PCR was done using a LightCycle480 SYBR I Master Mix (Roche Diagnostics, Germany) according to the manufacturer's instructions. qRT-PCR was performed with an initial denaturation at 95°C for 5 min followed by 40 cycles of denaturation at 95°C for 15 s, annealing and extension at 60°C for 30 s. GAPDH was used as an internal control. The primers were listed in Supplementary Table S2. Relative mRNA levels were calculated based on the Ct values and normalized using GAPDH expression, according to the equation: 2-ΔCt [ΔCt = Ct (Target) - Ct (GAPDH)]. All experiments were done in triplicate. A human apoptosis PCR array (CT Bioscience, PAI) was used to analyze the expression of key genes participated in ApoA-1 induced apoptosis, according to the manufacturer's instructions [20]. In brief, Huh7 cells were harvested after treatment with 50ng/ml ApoA-1 or ddH2O for 72 hours. Afterwards, total RNAs were extracted by Trizol regent (Lifetechnology, USA) and reverse transcripted into cDNA by Quntitect Reverse Transcription Kit (Qiagen, Germany).

### Statistical analysis

Statistical analyses were performed using SPSS 20.0 software (IBM, Chicago, IL, USA). Experimental values for continuous variables were expressed as the mean ± standard error of the mean. The chi-squared test, Fisher's exact probability tests and the Student's *t-*test were used as appropriate to evaluate the significance of differences in data between groups. If variances within groups were not homogeneous, the nonparametric Mann–Whitney test or the Wilcoxon signed-rank test was used. The relationships between serum ApoA-1 level and TTR and OS were analyzed using Kaplan–Meier survival curves and log-rank tests, respectively. Univariate and multivariate proportional analyses were performed using the Cox proportional hazard regression model and p<0.05 was considered statistically significant.

## SUPPLEMENTARY FIGURES AND TABLES







## Abbreviations

HCC: hepatocellular carcinoma
ApoA-1: Apolipoprotein A1
TTR: time to recurrence
OS: Overall survival
ROC: receiver operating characteristics
AFP: α-fetoprotein
BCLC: Barcelona Clinic Liver Cancer
CTCs: circulating tumor cells
PCR: polymerase chain reaction
