# Supplementary material for: Apolipoprotein A1: a novel serum biomarker for predicting the prognosis of hepatocellular carcinoma after curative resection
Source: Oncotarget. 2016 Sep 23;7(43):70654–68. doi: 10.18632/oncotarget.12203 (PMC5342581; doi:10.18632/oncotarget.12203)
Supplement: Supplementary file 1 [file oncotarget-07-70654-s001.pdf]

# Apolipoprotein A1: a novel serum biomarker for predicting the prognosis of hepatocellular carcinoma after curative resection

## SUPPLEMENTARY FIGURES AND TABLES

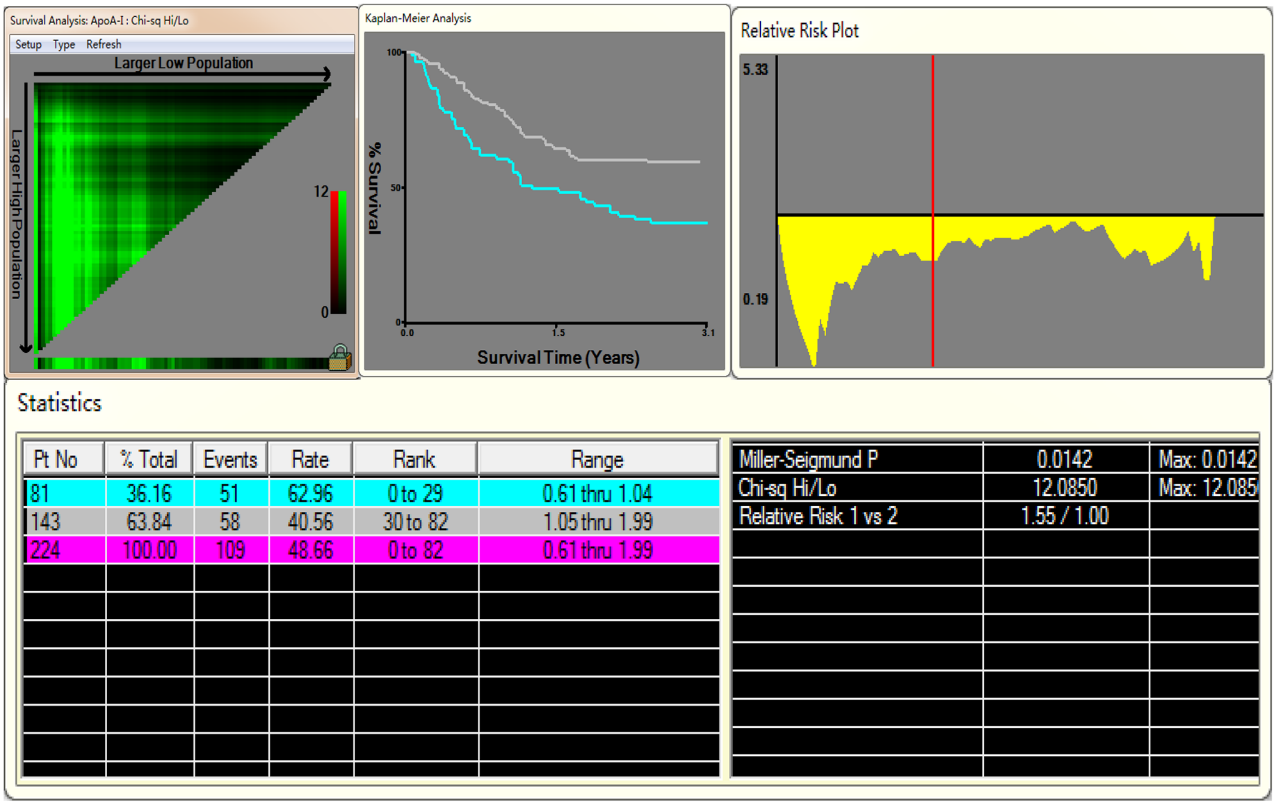


**Supplementary Figure S1: The optimal cutoff value of serum ApoA-1 levels was selected by X-tile3.6.1 software (Yale University, New Haven, CT, USA).**

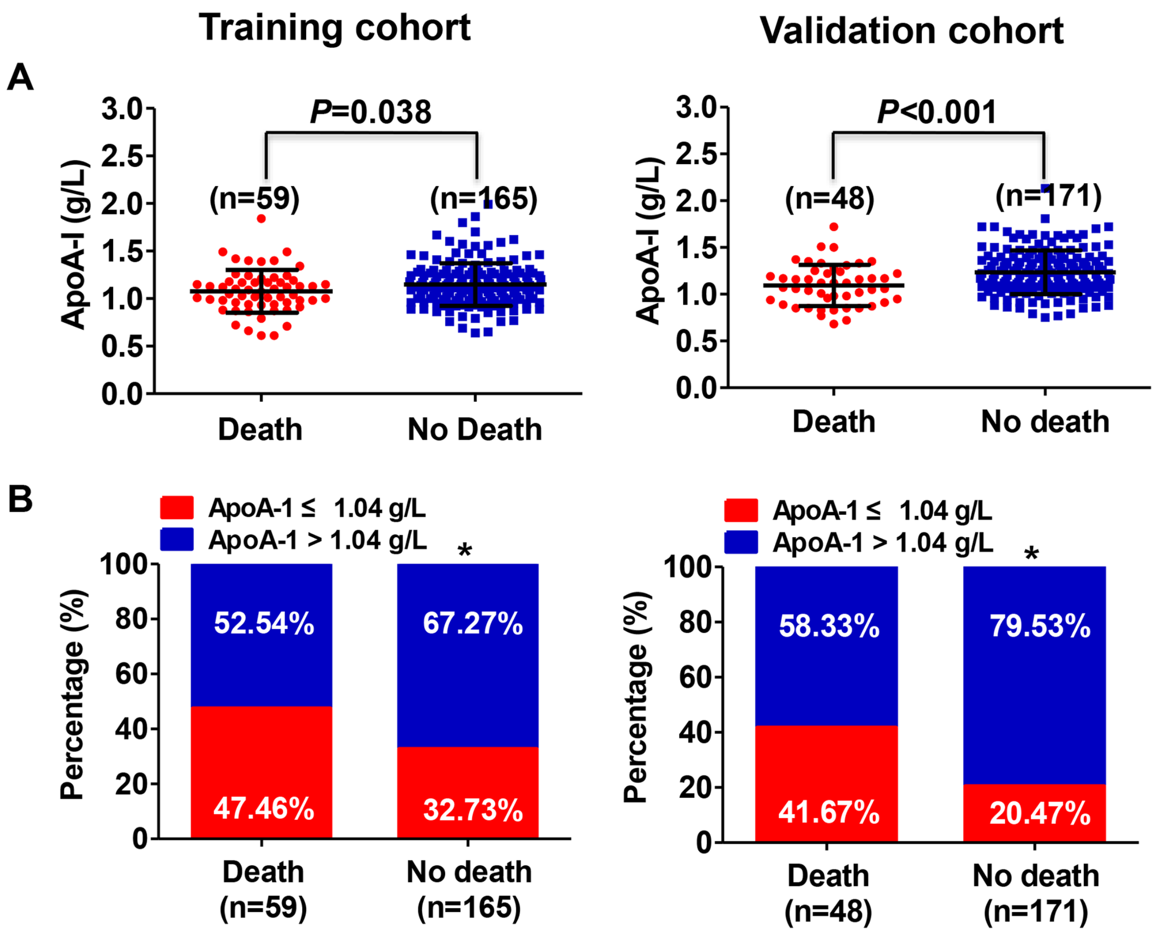


**Supplementary Figure S2: Survival significance of serum ApoA-1 levels in HCC patients underwent curative resection.** Distribution of serum ApoA-1 levels in dead and living patients from the training cohort (**A.** left) and from the validation cohort (**A.** right). Serum ApoA-1-positive rate in patients who died and living patients from the training cohort (**B.** left) and from the validation cohort (**B.** right) (*$P$ < 0.05).

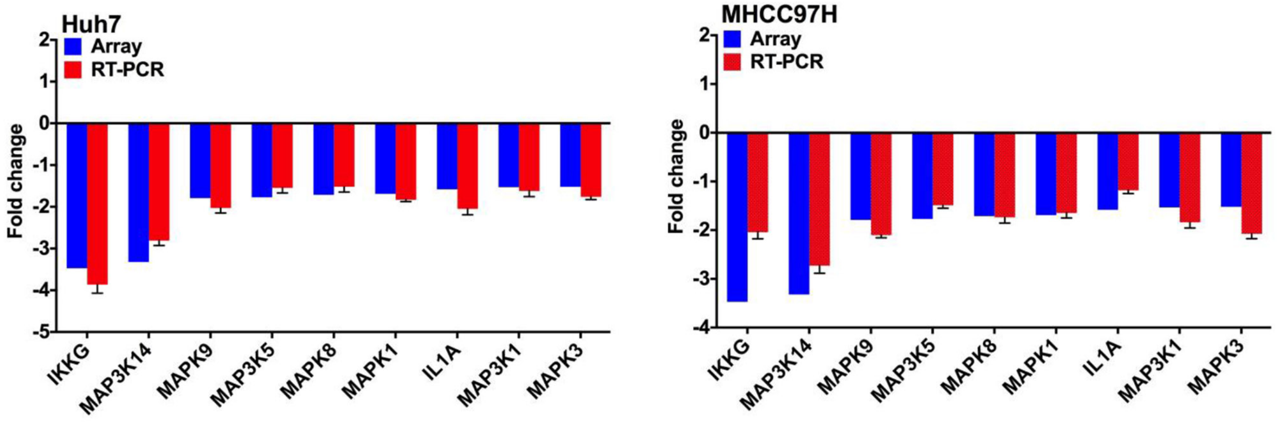


**Supplementary Figure S3: Validation of the results from PCR array.**
**Abbreviations: HCC, hepatocellular carcinoma; ApoA-1, Apolipoprotein A1.**

**Supplementary Table S1: Different expressed apoptosis-related genes in HCC cells after treating with ApoA-1.**

**See Supplementary File 1**

**Supplementary Table S2: Primers for RT-PCR.**

**See Supplementary File 2**
