# Supplementary material for: Apolipoprotein A1: a novel serum biomarker for predicting the prognosis of hepatocellular carcinoma after curative resection
Source: Oncotarget. 2016 Sep 23;7(43):70654–68. doi: 10.18632/oncotarget.12203 (PMC5342581; doi:10.18632/oncotarget.12203)
Supplement: Supplementary file 2 [file oncotarget-07-70654-s002.docx]

| **Supplementary Table 1 Different expressed apoptosis-related genes in HCC**  **cells after treating with ApoA-1** | | |
| --- | --- | --- |
| Symbol | Fold change | Expressed after ApoA1 treatment |
| CASP5 | 1.62 | Up |
| TNFRSF10B | 1.53 | Up |
| APAF1 | 1.52 | Up |
| IL7 | 1.40 | Up |
| PIK3CG | 1.33 | Up |
| IL6R | 1.27 | Up |
| PDCD1 | 1.27 | Up |
| CASP4 | 1.19 | Up |
| DAPK2 | 1.16 | Up |
| STAT1 | 1.15 | Up |
| MYD88 | 1.14 | Up |
| AKT3 | 1.13 | Up |
| CASP9 | 1.12 | Up |
| FADD | 1.11 | Up |
| MADD | 1.09 | Up |
| RELA | 1.09 | Up |
| PPP3CC | 1.08 | Up |
| TP53 | 1.08 | Up |
| CASP7 | 1.07 | Up |
| ERBB3 | 1.06 | Up |
| IL1B | 1.05 | Up |
| PIK3CA | 1.04 | Up |
| CASP6 | 1.04 | Up |
| IKBKB | 1.04 | Up |
| CD40LG | 1.01 | Up |
| AIFM1 | -1.01 | Down |
| IGF1 | -1.01 | Down |
| STAT5B | -1.02 | Down |
| NFKBIA | -1.04 | Down |
| AIFM2 | -1.05 | Down |
| PPP3R1 | -1.06 | Down |
| NFKB1 | -1.06 | Down |
| IL4 | -1.06 | Down |
| CASP3 | -1.07 | Down |
| AKT1 | -1.08 | Down |
| AKT2 | -1.08 | Down |
| CASP10 | -1.08 | Down |
| DFFA | -1.08 | Down |
| IGF1R | -1.08 | Down |
| STAT5A | -1.10 | Down |
| PDCD4 | -1.11 | Down |
| ACIN1 | -1.11 | Down |
| BIRC2 | -1.11 | Down |
| IRAK1 | -1.12 | Down |
| MAP3K10 | -1.12 | Down |
| DAPK1 | -1.13 | Down |
| E2F1 | -1.13 | Down |
| RIPK1 | -1.14 | Down |
| CDK1 | -1.14 | Down |
| TGFB1 | -1.17 | Down |
| CASP2 | -1.21 | Down |
| MAP3K11 | -1.21 | Down |
| PIK3R2 | -1.22 | Down |
| CD24 | -1.23 | Down |
| CIDEB | -1.23 | Down |
| JAK2 | -1.23 | Down |
| API5 | -1.25 | Down |
| EGFR | -1.27 | Down |
| CASP8 | -1.27 | Down |
| ENDOG | -1.33 | Down |
| TNFRSF10A | -1.37 | Down |
| DAPK3 | -1.43 | Down |
| XIAP | -1.44 | Down |
| BIRC3 | -1.50 | Down |
| MAPK3 | -1.52 | Down |
| MAP3K1 | -1.53 | Down |
| IL1A | -1.58 | Down |
| PDCD7 | -1.59 | Down |
| MAPK1 | -1.69 | Down |
| MAPK8 | -1.71 | Down |
| MAP3K5 | -1.77 | Down |
| MAPK9 | -1.79 | Down |
| MAP3K14 | -3.32 | Down |
| IKBKG | -3.47 | Down |

Abbreviations: HCC, hepatocellular carcinoma; ApoA-1, Apolipoprotein A1.
