# Supplementary material for: Apolipoprotein A1: a novel serum biomarker for predicting the prognosis of hepatocellular carcinoma after curative resection
Source: Oncotarget. 2016 Sep 23;7(43):70654–68. doi: 10.18632/oncotarget.12203 (PMC5342581; doi:10.18632/oncotarget.12203)
Supplement: Supplementary file 3 [file oncotarget-07-70654-s003.docx]

| Supplementary Table 2. Primers for RT-PCR | | |
| --- | --- | --- |
| Gene | Forward primer (5'-3') | Reverse primer 5'-3' |
| CLDN1 | TGCCTCAGTGGAAGATTTACTCC | TGGTGTTCAGATTCAGCAAGGA |
| CDH1 | GTAGGAAGGCACAGCCTGTC | CAGCAAGAGCAGCAGAATCA |
| FN | ACCTGGAGGAGACCACATGA | CCATCATCCAGCCTTGGTAG |
| CDH2 | GAGCATGCCAAGTTCCTGAT | TGGCCACTGTGCTTACTGAA |
| SNAIL | TCTGAGGCCAAGGATCTCCA | GTGGCTTCGGATGTGCATCT |
| VIM | CTGCAGGACTCGGTGGACTT | GAAGCGGTCATTCAGCTCCT |
| ZEB1 | GAGGATGACCTGCCAACAGA | TCCTGCTTCATCTGCCTGAG |
| MMP2 | CCAGATGTGGCCAACTACAA | GGCATCATCCACTGTCTCTG |
| MMP9 | CGAGACCGGTGAGCTGGATA | GTGGTGGTGCCACTTGAGGT |
| VEGFA | CCTTGCCTTGCTGCTCTACC | GCTGCGCTGATAGACATCCA |
| CASP5 | AACCGCAACTGCCTCAGTCT | GCCAAGGATGCTGGAGAGTC |
| APAF1 | CTCCATGATGGCATTCCTGT | CAGCCTGCCATTCCATGTAT |
| TNFRSF10B | CTGCACCAGGTGTGATTCAG | CGGCACATCTCAGGAGAATC |
| MAP3K1 | GCACCACCACTGCATGTCAA | TTGCTGCTGTGCAGCTCTGA |
| MAP3K5 | GGCCAACAACATCATCCTCT | AGTTCGGTTGCATGAGCTCT |
| MAP3K14 | AGGATGGAGGACAAGCAGAC | GCAGCTCCATGAAGATGTTG |
| MAPK1 | CTAACGTTCTGCACCGTGAC | TGGCCACATATTCTGTCAGG |
| MAPK3 | CTATGACCACGTGCGCAAGA | GACATTCTCATGGCGGAAGC |
| MAPK8 | GCAGAAGCAAGCGTGACAAC | GGCATCATAAGCTGCGCATA |
| MAPK9 | CGGACAGCGTGCACTAACTT | ACGGTCAGTGCCTTGGAATA |
| PDCD7 | TTATCTCCAAGCCGAGCACT | CCACACTCCTGGAGCATCTT |
| IL1A | TCACGGCTGCTGCATTACAT | GCAGCACTGGTTGGTCTTCA |
| BIRC3 | CCGTCAAGTTCAAGCCAGTT | CACGGCAGCATTAATCACAG |
| IKBKG  GAPDH | GCCTGGAGGAGAATCAAGAG  ATGGGGAAGGTGAAGGT | TCCTGGAACTTGCACATGAG  AAGCTTCCCGTTCTCAG |
| Abbreviations: CLDN1, claudin-1; CDH1, cadherin-1; FN, fibronectin; CDH2, cadherin-2; VIM, vimentin; MMP2, matrix metallopeptidase-2; MMP9, matrix metallopeptidase-9; VEGFA, vascular endothelial growth factor alpha; CASP5, caspase 5; APAF1, apoptotic peptidase activating factor-1; TNFRSF10B, tumor necrosis factor receptor superfamily 10b; MAP3K1, mitogen-activated protein kinase kinase kinase-1; MAP3K5, mitogen-activated protein kinase kinase kinase-5; MAP3K14, mitogen-activated protein kinase kinase kinase-14; MAPK1, mitogen-activated protein kinase-1; MAPK3, mitogen-activated protein kinase-3; MAPK8, mitogen-activated protein kinase-8; MAPK9, mitogen-activated protein kinase-9; PDCD7, programmed cell death-7; IL1A, interleukin-1, alpha; BIRC3, baculoviral IAP repeat containing-3; IKBKG, inhibitor of kappa light polypeptide gene enhancer In B-cells, kinase gamma; GAPDH, glyceraldehyde-3-phosphate dehydrogenase. | | |
